# Capturing Data on Antimicrobial Resistance Patterns and Trends in Use in Regions of Asia (CAPTURA)

**DOI:** 10.1093/cid/ciad567

**Published:** 2023-12-20

**Authors:** Marianne Holm, William R MacWright, Nimesh Poudyal, Alina Shaw, Hea Sun Joh, Patrick Gallagher, Jong-Hoon Kim, Affan Shaikh, Hye Jin Seo, Soo Young Kwon, Kristi Prifti, Brooke Dolabella, Ben E W Taylor, Corin Yeats, David M Aanensen, John Stelling, Florian Marks

**Affiliations:** International Vaccine Institute, Seoul, Republic of Korea; Public Health Surveillance Group LLC, Princeton, New Jersey, USA; International Vaccine Institute, Seoul, Republic of Korea; Public Health Surveillance Group LLC, Princeton, New Jersey, USA; International Vaccine Institute, Seoul, Republic of Korea; Public Health Surveillance Group LLC, Princeton, New Jersey, USA; International Vaccine Institute, Seoul, Republic of Korea; Public Health Surveillance Group LLC, Princeton, New Jersey, USA; International Vaccine Institute, Seoul, Republic of Korea; International Vaccine Institute, Seoul, Republic of Korea; International Vaccine Institute, Seoul, Republic of Korea; Public Health Surveillance Group LLC, Princeton, New Jersey, USA; Centre for Genomic Pathogen Surveillance, Big Data Institute, Oxford University, Oxford, United Kingdom; Centre for Genomic Pathogen Surveillance, Big Data Institute, Oxford University, Oxford, United Kingdom; Centre for Genomic Pathogen Surveillance, Big Data Institute, Oxford University, Oxford, United Kingdom; Brigham & Women's Hospital, Harvard Medical School, Boston, Massachusetts, USA; International Vaccine Institute, Seoul, Republic of Korea; Cambridge Institute of Therapeutic Immunology and Infectious Disease, University of Cambridge School of Clinical Medicine, Cambridge, United Kingdom; Heidelberg Institute of Global Health, University of Heidelberg, Heidelberg, Germany; Madagascar Institute for Vaccine Research, University of Antananarivo, Antananarivo, Madagascar

**Keywords:** antimicrobial resistance, surveillance, CAPTURA

## Abstract

**Background:**

In 2015, the UK government established the Fleming Fund with the aim to address critical gaps in surveillance of antimicrobial resistance (AMR) in low- and middle-income countries in Asia and Africa. Among a large portfolio of grants, the Capturing Data on Antimicrobial Resistance Patterns and Trends in Use in Regions of Asia (CAPTURA) project was awarded with the specific objective of expanding the volume of historical data on AMR, consumption (AMC), and use (AMU) in the human healthcare sector across 12 countries in South and Southeast Asia.

**Methods:**

Starting in early 2019, the CAPTURA consortium began working with local governments and >100 relevant data-holding facilities across the region to identify, assess for quality, prioritize, and subsequently retrieve data on AMR, AMC, and AMU. Relevant and shared data were collated and analyzed to provide local overviews for national stakeholders as well as regional context, wherever possible.

**Results:**

From the vast information resource generated on current surveillance capacity and data availability, the project has highlighted gaps and areas for quality improvement and supported comprehensive capacity-building activities to optimize local data-collection and -management practices.

**Conclusions:**

The project has paved the way for expansion of surveillance networks to include both the academic and private sector in several countries and has actively engaged in discussions to promote data sharing at the local, national, and regional levels. This paper describes the overarching approach to, and emerging lessons from, the CAPTURA project, and how it contributes to other ongoing efforts to strengthen national AMR surveillance in the region and globally.

## Teaser

This work presents an overall description of the CAPTURA project with emphasis on its scope and activities. We highlight how CAPTURA was able to engage in capacity-building activities, expanding antimicrobial resistance surveillance data, and supporting regional surveillance networks.

Antimicrobial resistance (AMR) is considered one of the biggest threats to global public health, with already measurable negative impacts for health and economy, particularly in low- and middle- income countries (LMICs) [1]. The independent “Review on Antimicrobial Resistance” (the “O’Neill report”), commissioned by the UK government in 2014 to inform and formulate key recommendations on how to tackle drug-resistant infections globally, estimated that if current trends continue unabated, by 2050 AMR will be the cause of 10 million annual deaths and a yearly cost of $100 trillion, with up to 90% of all deaths related to AMR occurring in Africa and Asia [2]. One of the major recommendations from this report was to improve the global collection of data on drug resistance and antimicrobial consumption (AMC) and to especially support lower income countries in achieving this [2].

In response to the early findings and recommendations by the O’Neill report, as well as to support the implementation of the 2015 World Health Organization (WHO) Global Action Plan on AMR [1], the UK government established the £265 million Fleming Fund with the overall aim to address critical gaps in AMR surveillance in LMICs in Asia and sub-Saharan Africa through a portfolio of country and regional grants, global projects, and fellowship schemes [3]. In 2018, the Fleming Fund regional round 1 grants were awarded with the specific objective of expanding the volume of historical and current data on AMR, AMC, and antimicrobial use (AMU) in the human healthcare sector [4].

An international consortium was awarded 2 out of 4 regional grants to work with 12 countries in South and South East Asia in conducting the Capturing Data on Antimicrobial Resistance Patterns and Trends in Use in Regions of Asia (CAPTURA) project, which started in early 2019 [5]. This paper describes the CAPTURA project, the emerging lessons on data identification and capture for national AMR surveillance planning in Asia, and how the project aligns with other related efforts both in the region and globally.

## PROJECT DESCRIPTION

### Project Scope

The CAPTURA project aimed to increase the volume of available AMR, AMC, and AMU data for decision making. Working with local governments as well as both private and public facilities, data, which are often unused, were identified and assessed for quality and availability. Relevant data were subsequently shared with CAPTURA, and collated and analyzed to provide a local overview as well as regional and interregional contexts, wherever possible.

The project focused on retrospective data collected between 1 January 2016 and 31 December 2019. The AMR data collected were bacteriology laboratory data particularly focusing on antimicrobial susceptibility test results on WHO priority bacterial pathogens (excluding *Mycobacterium tuberculosis*) [6]. Two types of AMC and one type of AMU data sources were included in CAPTURA and defined as follows—(1) Macro Antimicrobial Consumption: national-level AMC statistics such as total sales and import or export quantities in a country or region; (2) Micro Antimicrobial Consumption: records of antimicrobial procurement/supply/distribution at the district or facility level, but which do not hold data on individual dispensing; and (3) Antimicrobial Use: records of dispensed antimicrobials to individual patients.

### Project Design and Adaptive Approach

**Figure 1.**
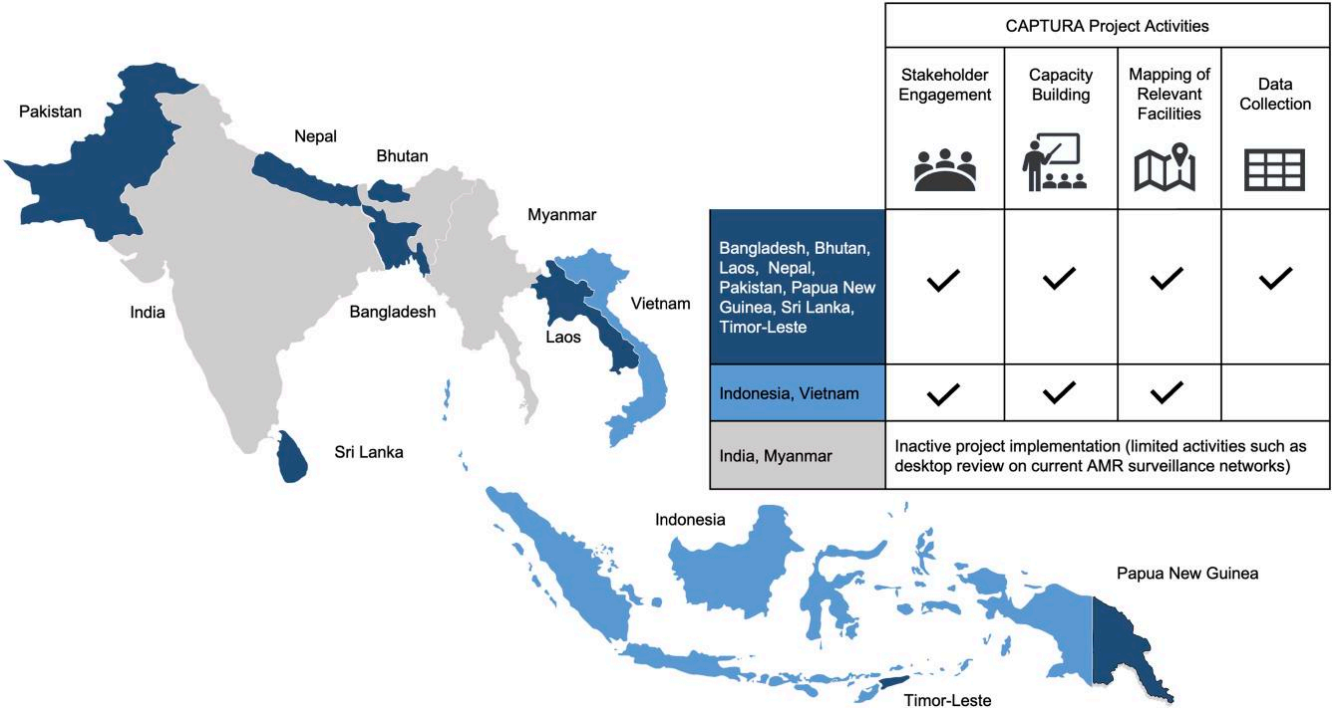
CAPTURA project phases and activities. Abbreviations: AMR, antimicrobial resistance; CAPTURA, Capturing Data on Antimicrobial Resistance Patterns and Trends in Use in Regions of Asia.

**Figure 2.**
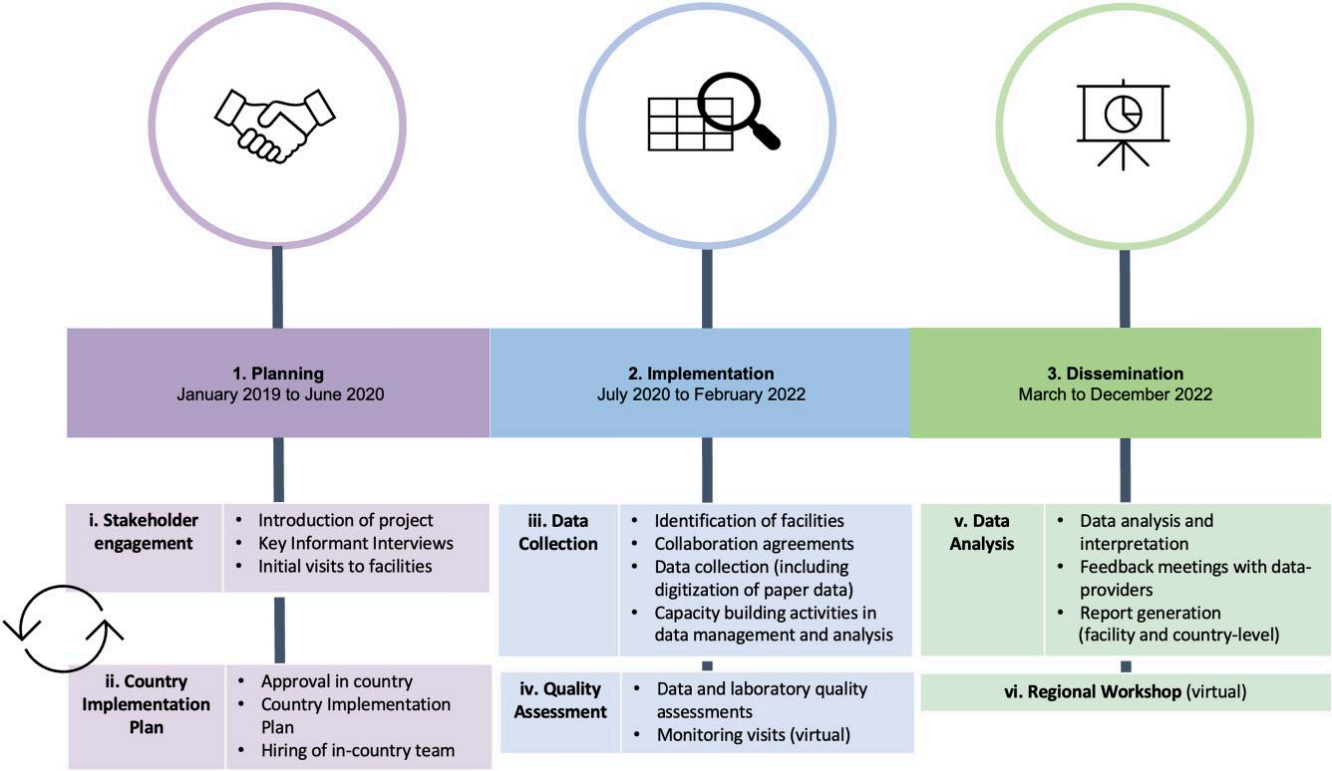
Three distinct phases of activities of the CAPTURA project.

The CAPTURA project was tasked to work across 12 countries in Asia, with active project implementation in 10 of these (see Figure 1). The Fleming Fund predefined these 12 countries as priority countries to implement the project. The project was conducted in three distinct phases of activities (Figure 2).

The initial phase of the project (January 2019–June 2020) included comprehensive scoping visits to 11 countries and engagement with key stakeholders. This included AMR coordinating committees within the relevant national ministries, drug administration and regulatory authorities, and designated national reference laboratories. We also engaged actively with other relevant stakeholders working within the field of AMR/AMC/AMU data capture in each country, including, among others, Fleming Fund country grantees [3] concurrently working to build local capacity for AMR surveillance across One Health sectors and WHO country offices involved in AMR-related activities. Based on the information gathered during these visits, we created individually tailored project implementation plans for each country (Figure 1).

From our initial scoping activities and when comparing with other previous attempts at mapping out the availability and status of AMR surveillance data [7], it was clear that desktop exercises have significant limitations in obtaining the relevant information. The high level of attention to, and already large number of initiatives in, combatting AMR across the countries requires careful coordination to enable synergies and avoid interference. Therefore, to ensure in-country presence and close communication with all relevant stakeholders, we engaged local teams of program coordinators and technical staff. Having these technical teams on the ground also facilitated remotely led implementation and ensured significant project progress even during the coronavirus disease 2019 (COVID-19) pandemic (see the article by Kwon et al in this supplement issue).

The initial engagement activities served to identify relevant facilities currently generating/holding data, including those participating in national surveillance systems as well as private laboratories or pharmacies across the country. Having identified relevant facilities, we administered questionnaires and conducted facility visits and laboratory assessments to evaluate the current capacity level in each facility for data management and analysis (see article by Joh et al in this supplement issue). All of this was crucial to establish collaborations and data-sharing agreements, as well as to identify relevant capacity-building needs across facilities. Significant efforts also went into understanding diverse data types and electronic formats to inform the prioritization and the optimal data retrieval processes. As illustrated in Figure 2, an important feedback loop between stakeholder engagement and implementation planning was included to ensure that activities were aligned with the expectations and needs of in-country stakeholders and, to some extent, individual facilities.

Due to the large variation between countries in existing capacities, and approaches to data collection, management, and use, a tailored approach/scope in each country was necessary to ensure local relevance and consideration of stakeholders’ preference (see Figure 1). Some countries preferred a scope of activities focused primarily on capacity building and/or supporting already existing data-generation efforts (Indonesia, Pakistan, Vietnam) with no or limited data retrieval and sharing. For example, in Indonesia, we supported capacity assessments in collaboration with the local AMR Coordinating Committee to assess data availability and map out facilities for potential future expansion of existing surveillance networks.

### Project Capacity Building

Training activities for in-country stakeholders were an integral part of the project and are described in more detail elsewhere (see article by Sujan et al in this supplement issue). In brief, across all countries, staff working with data management at facilities collaborating with CAPTURA received training in relevant software—in particular, the free WHONET software for management of microbiology laboratory data, as well as ad hoc support sessions for data curation, export, and analysis according to identified needs.

There was a wide range of data-management approaches in use across facilities and countries spanning from advanced Laboratory Information Management Systems (LIMS) to paper-based data collection, necessitating a tailored approach to capacity building to best fit the local context.

Training activities enabled facilities to correctly collate and curate data in a standardized format to help improve local data-management practices. This, in turn, could facilitate both current and future data sharing into a national data network (or build the foundation if such a network was not already in place) as well as enable local staff to become a knowledge resource for future trainings in data management and analysis in their respective facilities (and countries).

In the dissemination phase (March–December 2022), data analysis and visualization included the creation of local and regional displays of data-generating facilities with information on format, availability, and quality of data, and wherever possible, with links to retrospective isolate-level resistance and AMC/AMU data.

Final country reports for each country were generated and shared with national stakeholders. These reports summarized findings, experiences, and recommendations for future AMR surveillance data generation and management in alignment with national priorities (see articles by Poudyal et al and Prifti et al in this supplement issue). Going forward, the displays and evaluations of current data resources for AMR surveillance can hence be used by local governments in planning for the expansion of and improvement in current surveillance systems.

Finally, a regional workshop gathering local stakeholders from countries along with regional and global stakeholders to discuss CAPTURA project experiences, strategies to inform national efforts to combat AMR, and options for regional data sharing and collaborations in capacity building was held in June 2022 (see article by Joh et al in this supplement issue)

## CAPTURA IN CONTEXT

In recognition of the severity of AMR as a global health issue, concerted efforts have been initiated across the globe, including the creation of a Global Action Plan adopted by the World Health Assembly in 2015 [1] and further supported by the UN General Assembly in 2016 [8]. Despite substantial subsequent local commitment to implementation through the creation of National Action Plans [9], progress reports from the Interagency Coordination Group on Antimicrobial Resistance established by the UN suggest that there are still important gaps in the current global response [10] and that follow-through with planned activities is much lower than what was intended [11]. In particular, the lack in both quantity and quality of robust data has been repeatedly highlighted as one of the key challenges for meeting the requirements of the Global Action Plan and advancing surveillance efforts in LMICs [12–14].

### Expanding AMR Surveillance Data

One of the most notable achievements from the Global Action Plan on AMR has been the establishment of the Global Antimicrobial Resistance Surveillance System by WHO (GLASS), the first global effort to capture national AMR data in priority human pathogens [15]. The routine surveillance data stream of the GLASS system is composed of 2 separate modules, one for AMR [9] and another for AMC [16]. In the most recently published GLASS report released in December 2022, 87 countries (8 of these among the CAPTURA countries) submitted data for AMR. However, only 27 countries have so far enrolled in the AMC module (3 of these among the CAPTURA countries shared data) [17].

Although a major achievement for global AMR surveillance, some limitations of GLASS include the lack of complete data on several parameters including reliable data denominators, especially in LMICs. As such, the captured data are not yet sufficiently robust or representative to allow comparison between countries and regions or to be used for national treatment guidelines, identifying new threats, and outbreak detection. Even though in the most recent iteration of the GLASS report the scope has broadened and, for the first time, includes data on and analysis of resistance rates, differences in surveillance coverage and the representativeness of surveillance sites between countries and regions still render the interpretation of the global data limited [17].

The comprehensive work in CAPTURA of assessing current data-collection and data-management capacities and providing relevant support in improving these practices locally aims to not only develop the local capacity across facilities but also to enable countries to start contributing data and/or enhance the quality, quantity, granularity, and frequency of data submissions to their national-level surveillance systems as well as GLASS. In particular, the use of information technology for electronic data capture and collation is still limited [18, 19] and has been emphasized as an important tool enabling lower-income countries to “leapfrog” using these new technologies for surveillance improvement [20]. The capacity-building efforts in WHONET use for data management and the data retrieved through the CAPTURA project represent an additional and highly valuable resource for the countries in this regard as this enables more targeted approaches in building capacity and expansion of current national surveillance networks (see the article by Aboushady et al in this supplement issue).

### Supporting a Regional Surveillance Network

While discussions around data sharing are often considered challenging, from our recent experience with CAPTURA, most stakeholders are generally positive about the prospect of future national, and even regional, databases. However, there are often uncertainties about local data ownership and responsibilities and concerns about data access and use, which can hinder, restrict, or substantially slow down the decisions and approval processes on data-sharing agreements. This lack of clear data-access policies and resulting reporting delays has also been called to attention previously [14]. Through our recent work, it seems that the GLASS network has served to improve awareness on this issue across the Asian region, shown by the increase in countries enrolling and sharing data in GLASS between 2018 [21] and 2022 [17] (from 47 to 87 countries globally; 3 to 8 among the CAPTURA countries).

In addition to the global network established through GLASS, large regional AMR surveillance networks have been established in Europe (EARS-Net), Central Asia and Europe (CAESAR), and Latin America (ReLAVRA). However, no such systems are currently in place in Asia, which is a paradox, especially because Southeast Asia is the region with the highest proportion of countries reporting into GLASS (90%). It is our hope that the data-collation efforts through CAPTURA can not only help fill the current national data gaps but also, in synergy with other regional efforts [22], help catalyze the establishment of a similar, regional network in Asia. In 2020, WHO's Western Pacific Region established the Western Pacific Regional Antimicrobial Consumption Surveillance System (WPRACSS) to gather AMC data in the region, and this is a welcome foundation to build upon.

The national approach that the CAPTURA project has taken, partially mirroring the framework set out by GLASS, although more complex and time-consuming, can also provide substantial benefits to ensuring buy-in from laboratory up to national-level stakeholder, further promoting data quality and sharing, and ensuring the sustainability of a regional network [14]. In most project countries, CAPTURA has also considered and actively engaged with the commercial and/or private sector, which is an important element in understanding and controlling AMR and is currently underrepresented in many countries for both microbiology [23] as well as AMU and AMC data [7, 24]. Mapping and conducting assessments of additional facilities provides national stakeholders with an opportunity to build and expand on the current networks aiming for a more representative picture of local situations for future surveillance.

### Bridging Data Gaps

An early scoping report conducted to inform priorities for Fleming Fund activities identified a large number of separate initiatives dealing with surveillance and monitoring of AMR in LMICs, including 46 networks for bacterial pathogens, but also found poor coverage, particularly in the African and Asian regions [14]. Due to the state of existing health infrastructure in these regions, not only is the burden of AMR higher but also good-quality AMR data generation is particularly challenging due to the lack of adequate laboratory facilities (personnel, supplies, quality assurance) [25] and data management (personnel, equipment, modern data storage, and analysis architecture), ultimately affecting the establishment of efficient AMR surveillance programs [7].

Our initial activities in CAPTURA have confirmed these gaps and limitations, and in addition, we found a skew in focus and development between data collection and use between AMR, AMU, and AMC sources. AMR data collection has been in focus for longer in many countries, whereas only more recently have AMU and AMC data received much-needed attention. This was also seen in the very limited availability and retrieval of AMU data in CAPTURA. Similarly, even though AMC data have been collected through commercial entities (such as IQVIA, MIDAS®) [24], few countries consistently monitor the consumption or rational use of antimicrobials. Encouragingly, efforts to improve this area of data collection and use are underway in several countries and also globally through the WHO GLASS network AMC module [15].

Previous global efforts in assessing AMU, such as the Global Point Prevalence Survey (PPS) [26] conducted in 2015 (covering 303 facilities in 53 countries including 29 hospitals from 6 countries in Asia), were limited in terms of coverage and representativeness, especially for LMICs [27]. Similar challenges were seen in efforts trying to establish global or regional AMR data repositories [28]. In addition to not covering all countries in the Asian region, and particularly an underrepresentation of LMICs, this included relatively small, single data sources or small data subsets with very few numbers of isolates from national networks. They also lack contemporary data, with recent updates merely representing extrapolations/projections based on the previously compiled data [29]. Initiatives to engage and leverage commercially collected data have been established [30] and efforts are ongoing to making this openly available [31], with Pfizer's ATLASS® initiative in collaboration with the Open Data Institute currently a leading example for the industry [32]. Although information from such efforts will yield a higher granularity within individual datasets, and certainly a higher overall scientific value from the data, the overall quantity of data from each country in Asia is still limited [23].

Another consistent issue is the limited linkage between AMR (laboratory) and AMC/AMU (pharmacy) data as well as poor linkage to clinical information. Often, information on patient outcome or reliable denominators are missing [23]. As a response to this, more clinically oriented global initiatives to collect good evidence on AMR disease burden [33, 34] and case-based AMR surveillance [35] have been initiated.

The recent landmark publication on the global burden of AMR in 2019 by the Global Research on AntiMicrobial resistance (GRAM) project represents the most comprehensive, data-based estimations on human AMR to date [36]. While this work replaces earlier estimates largely based on modeling [2], another distressing finding, as highlighted by the authors, is the serious data gap from low- and middle-income settings and hence the skew towards data from high-income regions. There also remains a substantial scarcity in laboratory and linked clinical data; out of the 471 million individual records used in generation of the Global Burden of Disease (GBD) estimates only 511 870 patients’ records had known outcome and resistance information. It is hoped that in the next iteration of the GBD AMR study more substantial amounts and better-quality data from low-and middle-income settings in Africa and Asia can be incorporated.

The CAPTURA project made a concerted effort to bridge some of the identified gaps in data completeness and quality, which lie between the top-down approach taken by GLASS and the bottom-up approach by individual research initiatives aimed at answering questions on clinical outcomes. Nevertheless, limitations in data quality and/or representativeness are still prominent across the region. As such, we anticipate that the priority conclusions from much of the historical data will be about utilization of laboratory diagnostic services and testing practices, data quality, and management more so than on resistance epidemiology. However, understanding these results and using them to define the path towards better data is an essential step that presents countries with an opportunity to build or strengthen existing, national networks. The project has thus yielded valuable outputs for national and regional baselines on data generation, quality, and management and recommendations for building and improving prospective AMR surveillance systems across the Asian region. The logical extension would be to use these improvements in local data generation, and management to enable a strong regional surveillance network in the future.

## Notes

***Disclaimer*.** The views expressed here are those of the authors and not necessarily those of the UK Department of Health and Social Care or its management agent, Mott MacDonald; and, International Vaccine Institute.

***Author Contributions.*** M. H., W. R. M., J. M. S., D. M. A., and F. M. conceived the original concept and design of the CAPTURA program. M. H., W. R. M., N. P., A. G. S., H. S. J., P. F. G., J.-H. K., A. T. S., H. J. S., S. Y. K., D. B., and J. M. S. provided instrumental efforts in all in-country implementation activities. H. S. J., K. P., B. E. W. T., and C. Y. worked to compile and analyze data. M. H. wrote the manuscript. All authors provided critical revisions of the manuscript for important intellectual content. All authors read and approved the final version of this manuscript.

***Acknowledgments.*** The authors thank all their local CAPTURA team members who have made invaluable contributions to the conduct of site visits, trainings, and data collection activities for the project. They also thank all their external collaborators across the region who have provided valuable information during the in-country scoping visits as well as support and feedback during project implementation.

***Ethics statement.*** The Institutional Review Board (IRB) of the International Vaccine Institute (IVI) granted the project exemption from review since the project is not research involving human subjects, according to the definition in the IVI IRB SOP, D-RB-4-003.

***Supplement sponsorship.*** This article appears as part of the supplement “Capturing Data on Antimicrobial Resistance Patterns and Trends in Use in Regions of Asia (CAPTURA),” funded by the Department of Health and Social Care's Fleming Fund using UK aid.

***Financial support*.** This work was supported by the Fleming Fund regional grants (grant numbers FF10-135 and FF11-139). The Fleming Fund is a UK aid investment to support surveillance of antimicrobial resistance in low- and middle-income countries in Africa and Asia. The regional grants program is managed by Mott MacDonald Ltd, the Fleming Fund Grants Management Agent.
